# Understanding How the Brain Ensures Its Energy Supply

**DOI:** 10.3389/fnene.2012.00009

**Published:** 2012-08-20

**Authors:** Yuri Zilberter

**Affiliations:** ^1^Institut de Neuroscience des Systèmes, INSERM URM 1106, Aix-Marseille UniversitéMarseille, France

The theme of this research topic emerged in the hope of elucidating the mechanisms of energy supply dictated by costly neuronal activity. The versatility of the papers accepted to the topic is surprisingly broad. Three trends became evident, presumably reflecting the most vivid interests in the field: (1) the “*in vivo* versus *in vitro*” problem; (2) the role of particular energy substrates; and (3) the macro-level of energy homeostasis and how it applies to the dietary manipulations aimed at treatment of neurodegenerative disorders.

## Does the Brain Slice Tell the Truth, the Whole Truth, and Nothing But the Truth?

Brain slice studies have played an important role in gaining an understanding of fundamental neuronal, synaptic, and network traits but this preparation has never been free from serious limitations. Among them repeatedly mentioned are: absence of blood flow, diffusion peculiarities, inadequate oxygenation/metabolic support, and neuronal damage due to the slicing procedure (Ivanov and Zilberter, 2011; Bregestovski and Bernard, 2012; Hertz, 2012; Kann, 2012). On the other hand, the slice preparation is still considered an appropriate model even for the high energy-demanding modes of neuronal activity such as gamma oscillations, but only when care is taken to ensure an adequate energy supply to the slice (Kann, 2012).

The problem of extrapolation of the *in vitro* results to the *in vivo* reality seems to be something more than a mere methodological technicality. In the opinion paper titled “Excitatory GABA: how a correct observation may turn out to be an experimental artifact” (Bregestovski and Bernard, 2012), the history of the problem along with the latest discoveries and an impartial discussion shed light on one sensitive issue in developmental neuroscience – the meaning of observation on excitatory GABA in ontogenesis. Shetty et al. (2012), introducing in their paper a concept of Cerebral Metabolic Unit, discussed the short- and long-term metabolic responses to neuronal stimulation as well as the coordinated regulation of synaptic functions and blood flow, which is essential *in vivo* while completely absent *in vitro*. They also stressed the “observed predicted differences in metabolic function between *in vivo* and *in vitro* preparations.” In line with this notion, Ivanov and Zilberter (2011) demonstrated that neuronal activity *in vitro* requires considerably higher pO_2_ compared to that *in vivo*.

## The Role of Particular Energy Substrates

The ultimate recognized energy substrate synthesizing ATP in adults is glucose, but the view of its exact role seems to vary. The role of lactate, pyruvate, and beta-hydroxybutyrate is increasingly discussed (Schurr and Gozal, 2011; Hertz, 2012; Ruskin and Masino, 2012; Shetty et al., 2012). The fate of brain lactate *in vivo* is debated with opinions ranging from the hypothesis that lactate and not pyruvate is the end-product of aerobic glycolysis in the brain having a crucial role in coping with glutamate excitation (Schurr and Gozal, 2011) to the opinion that “exogenous lactate is not a necessary brain fuel” unable to protect from anoxic depolarization in slices (Hertz, 2012). On the other hand, in slices superfused with lactate-supplemented ACSF both oxidative metabolism and synaptic function were shown to be more efficient than in glucose-only based ACSF (Ivanov and Zilberter, 2011).

Neuronal energy demands are answered by ATP generation at a cost of energy substrate expenditure followed by metabolic signaling linking neuronal/glial activity to changes in blood flow (Shetty et al., 2012). This sequence of events is ensured by complex signaling mechanisms coordinating activities of neurons, astrocytes, and blood vessels. Molnar et al. (2011) showed a co-localization and important role of vascular and glial receptor recognizing both succinate and gamma-hydroxybutyrate, as well as the role of Ca^2+^ signaling related to energy metabolism. Urban et al. (2012), investigating BOLD signals, reported the existence of local neuronal circuits differentially controlling the blood flow in cortical barrel fields and the deeper areas.

## The Macro-Level of Energy Homeostasis and Dietary Manipulations

Kann (2012) in his review emphasized the exceptional vulnerability of higher brain functions to metabolic stresses. From the standpoint of the control systems theory, this vulnerability calls for reliable means to counteract the threat of metabolic failure – indeed, energy homeostasis is capable of employing alternative loops of control as soon as a safety margin is approached. This capability led to the conclusion that “it is possible that a final common neurometabolic pathway might be influenced by a variety of dietary interventions” aimed at restoring energy balance by shifting the failing pathway (usually glycolytic) to an alternative one (usually lipolytic/ketogenic; Rho and Stafstrom, 2012).

Indeed, a macro-nutrient ratio (a ratio of protein plus carbohydrate grams to fat grams) that increases chances of ketogenesis (while decreasing glycolytic flux) is shown to have favorable effects on metabolic profiles, thus exerting neuroprotective properties (Zilberter, 2011). The ketone-based metabolic mode is thought to operate by “providing elevated levels of high energy molecules (e.g., ATP, phosphocreatine) and increased capacity for energy generation (increased mitochondrial number)” and the mechanisms of its action may include increased levels of adenosine and GABA, decreased activity of glutamate as well as direct effects on ion channels (Ruskin and Masino, 2012). The assortment of neurodegenerative diseases responsive to metabolic intervention include “epilepsy, headache, neurotrauma, Alzheimer’s disease, Parkinson’s disease, sleep disorders, brain cancer, autism, pain, and multiple sclerosis” as listed by Rho and Stafstrom (2012).

Together, the articles comprising this Research Topic depict a complex intricately organized system with representative studies at both the macro- and micro-levels (e.g., involving molecular signaling pathways; Venkateswaran et al., 2012), which in the end ensures that the unparalleled energy demands of the brain are met in an urgent and extremely flexible manner – the trait yet to be fully understood and applied to the real life problems.
